# Experiences of alcohol-dependent elderly: grounded theory^*^

**DOI:** 10.1590/1980-220X-REEUSP-2022-0064en

**Published:** 2022-12-05

**Authors:** José Stéfano Faia Destro, Maria José Sanches Marin, Marcia Aparecida Padovan Otani, Jaqueline Dias do Nascimento Selleti, Elza de Fátima Ribeiro Higa

**Affiliations:** 1 Faculdade de Medicina de Marília, Programa de Mestrado Acadêmico em “Saúde e Envelhecimento”, Marília, SP, Brazil.; 2 Faculdade de Medicina de Marília, Marília, SP, Brazil.; 3 Universidade Federal do Paraná, Complexo Hospital de Clínicas, Núcleo de Estudos, Pesquisa e Extensão em Cuidado Humano de Enfermagem, Curitiba, PR, Brazil.

**Keywords:** Aged, Aging, Alcoholism, Grounded Theory, Nursing, Anciano, Envejecimiento, Alcoholismo, Teoría fundamentada, Enfermería, Idoso, Envelhecimento, Alcoolismo, Teoria Fundamentada, Enfermagem

## Abstract

**Objective:**

To interpret the experiences of alcohol-dependent elderly people.

**Method:**

Qualitative research developed through the theoretical and methodological assumptions of the Grounded Theory in the Straussian version. It was carried out in a small town in the mid-western region of the state of São Paulo. The selection was by theoretical sampling, totaling 25 participants from three sample groups. Semistructured interviews were conducted from March 2019 to January 2020.

**Results:**

The phenomenon “Experiencing alcohol dependence in old age”, is conditioned by the category “Initiating Alcohol Consumption”, are actions/interactions “Justifying alcohol consumption” and “Coping with alcohol treatment and abstinence” whose consequences are “Experiencing the harms of alcohol dependence” and “Expressing feelings”.

**Conclusion:**

It was evidenced that the elderly participants consider alcohol dependence as a way to deal with negative emotions, and, in this trajectory, they experience physical, mental, and social consequences. The elderly in abstinence express feelings of loneliness, regret, and desire for a life with quality, and indicate that behavioral change occurs through treatment and awareness of its harmful effects.

## RESUMO

**Objetivo:**

Interpretar as vivências dos idosos dependentes de álcool.

**Método:**

Pesquisa qualitativa desenvolvida por meio dos pressupostos teórico-metodológicos da Teoria Fundamentada dos Dados na versão straussiana. Foi realizada em um município de pequeno porte da região centro-oeste paulista. A seleção foi por amostragem teórica, totalizando 25 participantes a partir de três grupos amostrais. Foram conduzidas entrevistas semiestruturadas entre março de 2019 a janeiro de 2020.

**Resultados:**

O fenômeno “Vivenciando a dependência de álcool na velhice”, está condicionado pela categoria “Iniciando o consumo de Álcool”, são ações/interações “Justificando o consumo de álcool” e “Enfrentando o tratamento e a abstinência do álcool” cujas consequências são “Experienciando os danos da dependência de álcool” e “Expressando sentimentos”.

**Conclusão:**

Evidenciou-se que os idosos participantes consideram a dependência de álcool como forma de lidar com emoções negativas, e, nessa trajetória, experienciam consequências físicas, mentais e sociais. Os idosos em abstinência expressam sentimentos de solidão, arrependimento e desejo de uma vida com qualidade, e indicam que a mudança comportamental ocorre por meio do tratamento e conscientização de seus malefícios.

## RESUMEN

**Objetivo:**

Interpretar las experiencias de las personas mayores dependientes del alcohol.

**Método:**

Investigación cualitativa desarrollada a través de los supuestos teóricos y metodológicos de la Teoría Fundamentada en Datos en su versión straussiana. Se llevó a cabo en un municipio de pequeño tamaño de la región centro-oeste del Estado de São Paulo. La selección fue por muestreo teórico, totalizando 25 participantes de tres grupos de muestra. Las entrevistas semiestructuradas se realizaron entre marzo de 2019 y enero de 2020.

**Resultados:**

El fenómeno “Experimentar la dependencia del alcohol en la vejez”, está condicionado por la categoría “Iniciar el consumo de alcohol”, son acciones/interacciones “Justificar el consumo de alcohol” y “Afrontar el tratamiento del alcohol y la abstinencia” cuyas consecuencias son “Experimentar el daño de la dependencia del alcohol” y “Expresar sentimientos”.

**Conclusión:**

Se evidenció que los participantes de edad avanzada consideran la dependencia del alcohol como una forma de lidiar con las emociones negativas y, en esta trayectoria, experimentan consecuencias físicas, mentales y sociales. Los ancianos en abstinencia expresan sentimientos de soledad, arrepentimiento y deseo de una vida con calidad, e indican que el cambio de comportamiento se produce gracias al tratamiento y a la conciencia de sus efectos nocivos.

## INTRODUCTION

Population has been aging rapidly worldwide, and it is estimated that by 2060 the number of elderlies will quadruple and that the life expectancy of this population will exceed 80 years^(1)^. This change in the age profile brings new challenges, such as the increase in chronic health problems, among them, alcohol dependence, especially affecting developing countries like Brazil^(1,2)^. Alcohol is legally consumed, easily accessible, with low cost, popular and, along with tobacco, is the most commonly used substance by the elderly^(3)^.

Alcohol dependence among the elderly is an emerging issue, considered an invisible epidemic due to the large number of underreported cases. In Brazil, 2008 data from the first survey on alcohol consumption patterns in the elderly show that 12% were compulsive drinkers, 10.4% had subliminal dependence, and 2.9% were alcohol dependent^(2)^. In another study conducted with 111 elderly residents in the urban area of a city in the interior of São Paulo state, it was found that 22% of the respondents reported binge drinking and probable alcohol dependence^(1)^.

Events during the elderly’s life such as retirement, unemployment, loss of loved ones, loneliness, and social isolation may leave them vulnerable to adopting unhealthy habits and developing health problems, among them, alcohol dependence, characterized as a set of physiological, behavioral, and cognitive phenomena, in which the use of alcohol achieves a higher priority to the individual in detriment of other behaviors that previously had a higher value, as well as the inability to control its consumption despite the health consequences^(4,5)^.

With advancing age, the elderlies undergo biological changes, and their physiological tolerance to alcohol tends to decrease. Thus, alcohol dependence exposes the elderly to greater risks of worsening and developing physical, mental, social, and family problems^(6)^. It is worth adding that many older adults do not consider alcohol dependence as a risk behavior, either because of shame, fear, insecurity, or lifestyle, which hinders the identification of new cases and early interventions, contributing to increased hospital admissions, morbidity, and mortality^(7)^. This situation can be worsened when considering that the elderlies tend to seek treatment only when alcohol dependence causes severe physical or social consequences^(8)^.

The relationship between alcohol dependence, motivation, and commitment to change is complex in the cases of the elderly, as well as their preoccupation with drinking and their ability to control their consumption when compared to younger people^(8,9)^. Thus, it is of the utmost importance for the alcohol-dependent elderly to be treated in their entirety, emphasizing disease prevention and health promotion, with a view to reducing harm through their follow-up in health care networks. Data on alcohol dependence among the elderly in Brazil are scarce, which makes it a challenge for health professionals to investigate the impact that alcohol dependence has on their lives in physical, mental, and social dimensions^(1–10)^.

In this context, it is understood to be a complex problem with little visibility to health professionals and, more specifically, to nurses who can act in the promotion and rehabilitation, either through their actions in specialized mental health services or in primary health care. For this reason, it is relevant to deepen the knowledge, in order to adopt measures that meet the needs of these people. The present investigation is based on the following question: what have been the experiences of the elderly with alcohol dependence? The objective is to interpret the experiences of alcohol-dependent elderly people.

## METHOD

### Type of Study

This is a qualitative research study developed through the theoretical and methodological assumptions of Grounded Theory (GT) from the Straussian perspective. GT has been widely used in qualitative nursing research; its proposal centered on human action-interaction makes it relevant to the healthcare field, whose practices are based on the interactions between patients, families, and the work team. Therefore, it contributes to optimize the care provided to people from the understanding of perspectives and experiences lived in the specific health context, and seeks to develop a theory that emerges from data analysis, without the concern to confirm or confront a pre-existing theory^(11,12)^. To delineate the study with greater methodological rigor, the Consolidated Criteria for Reporting Qualitative Research (COREQ) checklist^(13)^ was employed.

### Setting

The study was developed in a municipality of the mid-western region of São Paulo with a population of approximately twenty-two thousand inhabitants, its Psychosocial Care Network (RAPS in Portuguese) consists of eight units of the Family Health Strategy (FHS), a mental health outpatient clinic and a Psychosocial Care Center (CAPS I) for the care of patients with alcohol dependence. Initially, data collection took place in the CAPS facilities and then in the FHS.

### Sample Definition

The sample definition was established by means of Theoretical Sampling. In this perspective, data collection starts with people or data sources considered relevant to answer the research question and its objectives. As the first data are analyzed, the next participants are selected. It is emphasized that the sample is not defined a priori, but during the course of the study, allowing the gaps in the emerging theory to be filled^(12)^. A total of 25 interviews were conducted

### Population

In accordance with the theoretical and methodological assumptions of GT, interviews were conducted with three sample groups, totaling 25 participants, in order to obtain data in an expanded way about the phenomenon under study. The first sample group was made up of 10 elderly people enrolled in CAPS, who met the inclusion criteria: 60 years or older, confirmed diagnosis of alcohol dependence, and cognitive ability to provide the required information. In the collection and analysis of the 10 interviews, it was verified that they had been abstinent for at least six months.

In a next step, there was the need to know if the elderly who still remained in excessive alcohol use could add new information to the phenomenon. The second sample group was then composed of six elders with the same characteristics as the previous group, but who maintained excessive alcohol consumption during the data collection stage, two of whom were enrolled in CAPS and the others were selected through contact with professionals from the HFS units. In these units, a meeting was held with the team of Community Health Agents (CHA) and indication of elderly people with the desired profile was requested.

From the collection and analysis of the interviews of the second sample group, it was possible to deduce that many data that emerged were associated with the family context and friendships of the elderly. Thus, with the purpose of elucidating knowledge about social interaction and friendships, expanding the understanding of the phenomenon, the third sample group was formed, composed of seven family members and two friends of the elderly interviewed, totaling nine participants. The collection was closed with the third sample group, respecting the criteria of theoretical saturation, when no additional relevant data was found, when the findings filled the gaps of knowledge, which allowed the interpretation of the phenomenon and it was possible to establish the relationships between the categories^(11)^.

### Data Collection and Analysis

Data collection and analysis were performed by means of the deductive inductive process, where deduction allows for the construction of hypotheses, while induction enables the apprehension of implications originating from the hypotheses in order to qualify or refute them^(12)^. Data were collected during the period March 2019 to January 2020 by the principal researcher, who is a master’s degree student and has received training in in-depth interview technique. The interviews with the first sample group were conducted on the premises of the CAPS, and the elderly were approached as they attended the facility for care. After the subjects expressed their agreement to participate, a time of their convenience was scheduled for the interview.

A script was prepared to be applied on this group. It contained the identification data and the following questions: How was your first contact with alcohol? What was the importance of alcohol in your life? What are the consequences of alcohol dependence? Please tell us about the influence of alcohol consumption on family relationships. What led you to seek treatment and what are the benefits of abstinence? Please tell us about your feelings regarding alcohol consumption and your expectations for the future.

In the second group, the contact with two elderly people was made at CAPS. For the others, home visits were made, accompanied by the CHAs, and the collection was done at home. For this sample group, the following questions were asked: How was your first contact with alcohol? Why do you continue to consume alcohol? What is the importance of alcohol in your life? What is your family context like? What are the possible consequences you experience as a result of excessive alcohol consumption? Tell us about your feelings and your expectations for the future.

For the third sample group, besides the sociodemographic data collected from family members and friends, the interview script for the family members included questions about living with the alcohol-dependent elderly individual, the consequences for the elderly individual and family members, and the feelings expressed. The friends of the elderly were questioned about their social interaction and friendship relations. There were no refusals to participate in the research, and each participant was interviewed for an average duration of 30 minutes, conducted individually, in a calm environment, free of interruptions, and with guaranteed privacy for the interviewee. The interviews were audio recorded and later transcribed.

Data analysis in the Straussian approach to GT is organized in three steps: open coding, axial coding, and integration. In open coding, the researcher must fixate on the data collected, examining them rigorously line by line, comparing and contextualizing them with words that convey action, enabling the identification of substantive codes. In the axial codification, the data that were separated in the previous step are regrouped, allowing the emergence of categories that will be reorganized and related to their subcategories, establishing connections among them and generating more precise and complete explanations about the phenomenon^(11,12)^.

To organize such connections, an analytical tool called paradigmatic model is used that helps to gather and order the data so that the structure and process are integrated, consisting of three components: conditions, actions/interactions and consequences^(12)^. Conditions refer to the reasons or explanations given by people of why and how they respond to events and problem situations. Actions/interactions correspond to the meanings assigned by people to events and problem situations that have occurred in their lives and actions/interactions taken to manage the problem or achieve the goal. The consequences component refers to the outcomes resulting from the actions and interactions, these consequences can be physical, psychological or social^(11)^.

The last step is integration. The categories and subcategories are organized around a central explanatory concept that represents the main theme of the research. At the end of the coding steps, the generated theory is reorganized according to the elements of the paradigmatic model^(11,12)^. All steps of the data analysis had the participation and consensus of the authors. The theoretical model was first validated by the research participants through individual meetings at the CAPS facilities and home visits in which the theoretical model was presented and the results of this research were explained. There was confirmation from the participants that those results express their experience and translate their daily lives. Afterwards, the work was submitted to a panel of experts in qualitative research and experience with GT, who validated the results.

### Ethical Aspects

This research respected all ethical requirements provided in Resolution 466/12 of the National Health Council (CNS). The research obtained approval from the Research Ethics Committee of the proposing institution under consubstantiated opinion no. 3,325,707, dated April 1, 2019. The participation of the interviewees occurred after signing the Informed Consent Form on a voluntary basis. The participants were identified as follows: seniors in abstinence with the letters IA, seniors in excessive alcohol use with the letters IU, family members of the seniors with the letter F, and the friends of the interviewees with the letter A, followed by a number referring to the order in which the interviews were conducted.

## RESULTS

Among the study participants, the first and second sample groups consisted of 10 and six elders, respectively. The majority are male (13), average age of 66 years, eight married, five divorced, two single, and one separated. Six elderlies are unemployed, five are retired and five are self-employed, with an average monthly income of two minimum wages and no income. Nine of the 16 elderly people interviewed have incomplete elementary school education, four have completed high school, and three have completed elementary school.

About the third sample group, among the seven family members of the elderly, the totality is female, aged between 41 and 71, and married. Three are self-employed, two retired, one is a businesswoman and one is unemployed, with monthly incomes varying between over three minimum wages and no income. Regarding the level of education, three have completed elementary school, three have incomplete elementary school, and one has completed high school. The two friends, both male, aged 52 and 53, one married, living with his wife, self-employed, with a monthly income above three minimum wages, and with a complete high school education. The other, divorced, lives with his mother, works as a civil servant, monthly income above two minimum wages, with complete college education.

**Figure 1.**
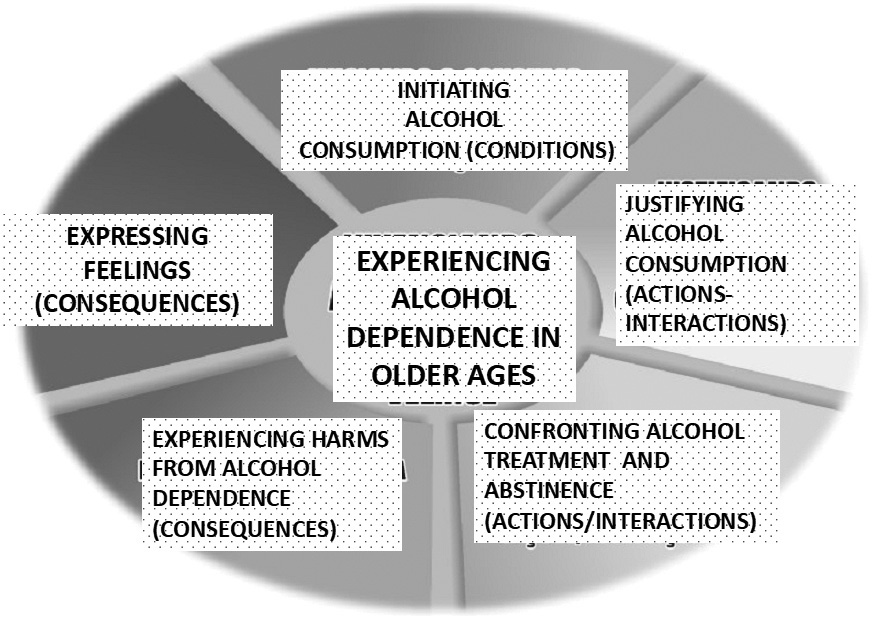
Theoretical model “Experiencing alcohol dependence in old age” – Marília, SP, Brazil, 2021.

From the analysis of the data obtained in the interviews, we structured Figure 1, which presents the theoretical model “Experiencing alcohol dependence in old age”.

The paradigmatic model allowed to systematize the data relating its components to the phenomenon “Experiencing alcohol dependence in old age”, being conditioned to the onset of alcohol consumption in the lives of the elderly, and actions-interactions are the justifications presented for consumption and coping with treatment and abstinence from alcohol. These actions-interactions have, as consequences, the damages resulting from alcohol dependence, such as harm to physical, mental and social health, and the expression of feelings like regret and loneliness.

The conditions component of the phenomenon is characterized by the category “Initiating drinking”. The beginning of alcohol consumption usually occurs in adolescence; however, in some cases, its onset can be late, being associated with events that occurred during life, such as retirement, having a negative impact on the life of the elderly.

*17 years old, I started because, right, youthful curiosity right (…) (AI 5)*

*When he retired (…) after he started to stay more at home, then he started to drink straight, every day and every hour, (…) he had idle time. (F 2)*

Facing the conditions experienced, the category “Justifying alcohol consumption” is characterized as one of the actions/interactions, in which the participants justify the consumption as a resource to deal with situations that cause suffering, which they could not manage with abstinence. The use of alcohol is directly associated with the feeling of well-being, pleasure, and increased willingness to work that are only experienced under the effect of alcohol.

*I drank to escape a little from reality (…) I felt overwhelmed, I drank and relaxed (…) when I didn’t drink, I was sad and cried. (AI 11)*

*When I drank, I was happy; when I didn’t drink, I was withered (…) when I didn’t drink, it seemed that something was missing (…). (AI 6)*

*I used to drink ‘pinga* ^*1*^ *’ to work, that gave me courage! (…) when I didn’t have anything to drink, I felt like going away, when I drank, my work was more productive. (AI 8)*

The alcohol-dependent elderly people who continue to consume alcohol deny that it has caused any harm and, because of that, they do not recognize the need to seek help.

*(…) I never felt anything, the doctor only said that I had a small spot on my lung from smoking, and a small spot on my liver from drinking, and that’s it! (IU 2)*

Another category associated with actions/interactions is “Confronting alcohol treatment and abstinence”. Through treatment and abstinence from alcohol, the elderly experience physical, financial, and social benefits, and become aware of the consequences of alcohol dependence, and that the resumption of its consumption is a path of no return because they are no longer physically and emotionally able to bear such a burden.

*Health is great, (…) my health is different, you don’t even see me sneeze. (AI 3)*

*After I stopped drinking, even money is left over (…) I feel different, I don’t know how to explain it, I am calm, I don’t have any more blood pressure problems. (AI 6)*

*(…) I want to stay strong, because if I fall again to drinking at this age and with these diseases, maybe I won’t be able to get hospitalization and come back alive. (AI 9)*

Two categories present themselves as consequences, the first being “Experiencing harms from alcohol dependence”. The consequences of alcohol dependence affected the physical, mental and social health and family context of the elderly, in addition to financial difficulties.

*(…) his legs started getting stiff, he can no longer eat alone, he only takes a shower sitting down, he doesn’t stand up anymore, I don’t see any improvement, my father is getting worse (…). (F 7)*

*(…) before he only walked around drunk, acting as a puppet for the gang, the guys made fun of us: “There, the drunk guy! “. It is annoying and shameful (AI 9)*

*(…) there were times that I would stop paying a bill to pay the bar debt (…) sometimes, I would stop buying something for home to buy a drink. (IA 6)*

*Nowadays, the coexistence is not good either because of these drinks that he takes, so I sleep in one room with my grandson and he sleeps in another, this has been going on for about 7 years. (F 6)*

Another category related to the consequences component is “Expressing feelings”. The feelings expressed only by the participants in abstinence are related to loneliness, arising from the affective distance from family and friends, regret for actions performed under the influence of alcohol, and the desire to improve the quality of life that was affected by alcohol dependence.

*I feel loneliness, I miss my children, many of my friends have died (…) contempt is the worst thing in the world, contempt is sad. (AI 4)*

*I regret not having a better house and a better car (…) having lost my job and two marriages (…). I regret not having a better life. (AI 9)*

*(…) I want to improve more (…) I want to last a few more years, but whatever I can do to improve my health, I will do, yes, God willing! (AI 6)*

## DISCUSSION

The first contact with alcohol may occur early because of the search for the new and the desire for new experiences, better interpersonal relationships, or even individual characteristics such as insecurity^(14)^. The beginning of alcohol intake may, however, occur later, as a resource to cope with stressful events such as retirement, mourning, loneliness, social isolation and financial difficulties, making the elderly susceptible to the development of alcohol dependence^(5)^.

In the theoretical model presented, alcohol consumption is related to the expression of positive feelings. The effects of alcohol on the body enhance feelings of pleasure, promoting socialization, social disinhibition, and positive effects on mood^(7–15)^. The choice of alcohol consumption ultimately depends on the free will of the subject, which can be understood from his intentionality, based on his life history. Although free will derives from a cause, even if it is self-will, it is still influenced by intersubjectivity and context in which the individual is inserted^(15)^. Thus, the elderly may consume alcohol as part of their social life to obtain fun and pleasure, or adopt such habit from people close to them who evaluate consumption as a social norm^(5)^.

In view of the positive effects of alcohol, many dependent elder people underestimate the consequences of its consumption, their ingestion becomes uncontrollable, triggering dependence and the need for daily alcohol intake. Thus, they prioritize its use over other activities, minimize its effect, refuse to seek specialized assistance, and allege a false sense of control over their consumption^(7)^.

However, it is found that elderlies are motivated to reduce consumption or totally abstain from alcohol when they understand that drinking can affect their health and hinder control in maintaining their quality of life and longevity^(16)^. According to the results of the present study, the alcohol-dependent elderly, when they start treatment and maintain abstinence, can experience its benefits. Individuals in abstinence from alcohol, show improvement in health conditions and ability to work, financial situation, concentration and energy levels, and confidence in resisting alcohol^(17)^.

The elderly participants reported that through treatment, awareness of the harm of alcohol dependence emerges. From negative experiences and the perception of the vulnerability of their health, comes the understanding, the confrontation of what they want and what they no longer want to experience, emerging the understanding, the acceptance of the disease and recognizing the need for treatment^(18)^. It is noteworthy, however, that for the elderly, the perception of their health status involves issues of social nature and its relationship with the use of alcohol, which can be both in strength as in weakness in the search for treatment.

Therefore, among the interviewed elderly who maintain excessive alcohol consumption, they fail to recognize the disease or present a desire to change. The lack of perception of the need for treatment is a complex behavior, related to personal attitudes and beliefs and social patterns that negatively influence the search for assistance^(19)^.

Owing to changes associated with aging, the elderlies are more vulnerable to the effects of alcohol compared to younger people. Alcohol reaches higher blood levels, leading to intoxication even in small amounts consumed. In addition, sensitivity to alcohol increases, as, tolerance decreases^(20)^. Excessive alcohol consumption is one of the major risk factors for the development of a number of health problems. Such conditions may be exacerbated in the case of older adults with poor physical health and who take medications that may interact with alcohol^(5–20)^.

The interviewees’ reports highlight the relationship of alcohol dependence with family conflicts, leading them to experience a family context, marked by fights and affective distancing, characterizing the family as a deteriorated unit^(21)^. Alcohol dependence is associated with divorce, especially among the elderly, due to increased stress, which can impact the physical health of the individual. However, finding data on the elderly population, becomes a challenge due to the underreporting of cases and understudied theme^(5)^.

Among other consequences pointed out, the alcohol-dependent elderlies suffer from the stigma of the disease. Alcohol dependence is the fourth most stigmatized health condition worldwide, making them a vulnerable group of the population, contributing to the deterioration of social relationships and loneliness^(22)^. According to abstinence respondents, alcohol dependence has an association with loneliness, because when abstaining from alcohol, it is necessary to avoid relationships that may stimulate its consumption, which may mean, a redefinition of the social cycle, seeking to connect to people who support their recovery. This process probably increases the risk of loneliness. Data show an association between occasional alcohol consumption and decreased loneliness and stress, as well as increased self-esteem and optimism, by providing an environment of social contact and bonding among users, reducing social isolation and decreasing feelings of loneliness^(22,23)^.

The feeling of guilt or regret is only expressed after the abstinent individual reflects on what he or she has done. Those who persist in using alcohol, even acknowledging the behavioral changes and changes in the family context, marked by aggressiveness and fights, usually do not express guilt or regret by minimizing their consumption^(24)^.

It is essential to provide specialized assistance to the alcohol-dependent elderly. For this purpose, CAPS stands out as a reference service in the treatment of alcohol dependence in the adult population, by playing the strategic role of articulating the network and the mental health policy in the territories. CAPS follows the logic of community care, focused not only on the treatment of users and alcohol abstinence, but also on their family, social, and community reinsertion^(25)^. That said, the nursing professional may be considered as one of the main providers of assistance to alcoholics, because through his work process, he is closer to this clientele. It is imperative to rethink the theoretical/practical teaching during academic training, and it should be anchored in the current mental health policy, with the deconstruction of prejudices and breaking of traditional practices and conceptions, as well as stimulating continued and permanent education in the spaces of mental health care^(26)^.

This study’s limitation resides in that it was conducted in only one treatment site for alcohol dependence, but it does not invalidate its results and may contribute to the development of future studies.

## CONCLUSION

When interpreting the experience of alcohol-dependent elderly individuals, the theoretical and methodological assumptions of the GT allowed for the deepening and interpretation of the main aspects of the phenomenon in question, through intense and constant interaction with the data collected, reflections, and comparisons made, being of utmost importance to explore the richness and uniqueness of the experiences of alcohol-dependent elderly individuals.

The construction of the theoretical model represents an important contribution with regard to alcohol dependence among the elderly by analyzing and interpreting the problem from the point of view of the subjects themselves. It was evidenced that the onset of alcohol consumption occurs at different moments, either early or late. It was unanimous, in the experience of the elderly, to relate alcohol dependence as a means to deal with negative emotions and, little by little, assuming a central role in their lives. Faced with this meaning, modifying such behavior becomes a challenge, the behavioral change occurs through treatment and awareness of the harmful effects of alcohol dependence. In the trajectory of alcohol dependence, the elderly experience physical, mental, and social consequences that are often irreversible. The elderly in abstinence, despite experiencing its benefits, express suffering and feelings of loneliness due to the breakdown of the affective bond with loved ones, regret for their actions while consuming alcohol, in addition to the desire to live longer and with quality.
